# Trends in Industry-Sponsored Research Payments to Physician Principal Investigators

**DOI:** 10.1001/jamanetworkopen.2024.12432

**Published:** 2024-05-16

**Authors:** Zhuo T. Su, Zeyad Hammadeh, Joseph G. Cheaib, Yuezhou Jing, Bruce J. Trock, Misop Han

**Affiliations:** 1 James Buchanan Brady Urological Institute, Johns Hopkins University School of Medicine, Baltimore, Maryland

## Abstract

This cohort study investigates trends in total and per-physician industry-sponsored research payments to physician principal investigators from 2015 to 2022.

## Introduction

After passage of the Physician Payments Sunshine Act (PPSA), the Open Payments program (OPP) was launched in 2013 to increase transparency of physician-industry financial relationships.^1^ Because research payments constitute the largest payment category in the OPP,^2,3^ we characterized trends in industry-sponsored research payments (ISRPs).

## Methods

This cohort study follows the Strengthening the Reporting of Observational Studies in Epidemiology (STROBE) reporting guideline. We used OPP data^4^ to identify ISRPs inflation-adjusted to 2022 US $ values during 2015 to 2022. In the OPP, covered recipients include teaching hospitals, physicians, and advanced practice practitioners not employed by applicable organizations reporting payments. Noncovered entities (NCEs) are organizations that do not meet the OPP definition of covered recipients. ISRPs to NCEs are reportable if a covered physician is a principal investigator (PI).^4^ We analyzed ISPRs to NCEs with physician PIs because these constituted most ISRPs by value (>70%). Where multiple (up to 5 could be listed per ISRP) PIs were listed for an ISRP, we attributed the full amount of that ISRP to the primary PI given a lack of disclosure requirements about fund allocations within an ISRP. This attribution avoided double counting of the same ISRP. We tested trends in total values using linear regression and trends in per-physician ISRPs using generalized linear models with a γ distribution to account for physician effects, with a 2-sided α < .05 applied. We used Stata statistical software version 18.0 (StataCorp).

## Results

Overall, ISRPs increased 20.0%, from $6.32 billion in 2015 to $7.58 billion in 2022 (*P* = .01). ISRPs to NCEs increased 19.1%, from $4.98 billion in 2015 to $5.93 billion in 2022 (*P* = .06), and the trend was not statistically significant; the total in 2022 accounted for 47.1% of all Open Payments ($12.58 billion) that year. In contrast, ISRPs paid directly to covered physicians decreased 61.7%, from $179.5 million to $68.8 million (*P* < .001) (Table 1).

**Table 1.** Research Payments From Industry to Covered Recipients

Recipient type^a^	Annual research payment values, 1 000 000 US $ (% of total research payment values)^b^								Overall % change	*P* value^c^
	2015	2016	2017	2018	2019	2020	2021	2022		
Covered teaching hospital^d^	1158.7 (18.3)	1331.1 (20.3)	1457.3 (21.6)	1637.5 (23.3)	1714.4 (25.7)	1710.8 (25.4)	1703.5 (22.1)	1583.0 (20.9)	36.6	.02
Covered physician	179.5 (2.8)	180.0 (2.7)	126.7 (1.9)	124.4 (1.8)	105.8 (1.6)	83.6 (1.2)	79.4 (1.0)	68.8 (0.9)	−61.7	<.001
Covered nonphysician practitioner^e^	NA	NA	NA	NA	NA	NA	0.4 (0.01)	0.4 (0.01)	−5.8	NA
NCE	4978.7 (78.8)	5056.2 (76.9)	5159.7 (76.5)	5252.4 (74.9)	4856.3 (72.7)	4947.9 (73.3)	5910.4 (76.8)	5931.6 (78.2)	19.1	.06
Noncovered individual	5.0 (0.1)	4.7 (0.1)	3.7 (0.1)	0.7 (0.01)	0.6 (0.01)	6.1 (0.1)	1.0 (0.01)	0.3 (0.004)	−93.4	.16
Total	6321.9	6572.0	6747.4	7015.1	6677.1	6748.4	7694.7	7584.2	20.0	.01

Abbreviations: NA, not applicable; NCE, noncovered entity.

^a^ Definitions of recipients are from the Centers for Medicare & Medicaid Services Open Payments program (eMethods in Supplement 1).

^b^ All payment values were inflation-adjusted to 2022 US dollars using the Consumer Price Index for All Urban Consumers: Medical Care in US City Average.

^c^ *P* values were obtained from linear regression.

^d^ See eMethods in Supplement 1 for the definition of teaching hospitals.

^e^ Reporting of payments to covered nonphysician practitioners started in 2021.

ISRPs to NCEs with a physician PI increased 23.0%, from $4.52 billion in 2015 to $5.55 billion in 2022 (*P* = .03), accounting for 44.1% of all Open Payments. General internists had the highest annual median ISRPs per PI (Table 2). In 2022, ISRPs were made to NCEs with 21 518 physicians as primary PIs, accounting for 2.0% of US physicians. The maximum ISRP received by an NCE with a physician PI was $89.5 million.

**Table 2.** Research Payments From Industry to NCEs With Physician PI

Measure^a^	Year								Overall change, %	*P* value^b^
	2015	2016	2017	2018	2019	2020	2021	2022		
**All physicians**										
Physicians as primary PI for NCEs receiving payments, No.	26 126	25 808	25 690	25 373	24 183	23 126	22 772	21 518	−17.6	<.001
Payment value, million US $	4517.6	4609.6	4772.8	4893.1	4527.2	4636.3	5537.1	5554.7	23.0	.03
Payment value per PI, median (IQR), US $	27 750 (5619-115 559)	30 426 (6931-124 241)	30 297 (6663-127 008)	32 193 (7375-131 598)	32 240 (7314-136 699)	31 276 (7108-128 447)	31 049 (7374-130 889)	32 679 (7932-134 456)	17.8	<.001
**General internists** ^c^										
Physicians as primary PI for NCEs receiving payments, No. (% of total)	4871 (18.6)	4958 (19.2)	5129 (20.0)	5060 (19.9)	4757 (19.7)	4639 (20.1)	4771 (21.0)	4660 (21.7)	−4.3	.07
Payment value, million US $ (% of total)	1135.8 (25.1)	1175.3 (25.5)	1134.9 (23.8)	1063.7 (21.7)	1044.8 (23.1)	1162.4 (25.1)	1795.4 (32.4)	1941.0 (34.9)	70.9	.04
Payment value per PI, median (IQR), US $	41 908 (7633-175 897)	41 401 (8892-176 068)	39 106 (7668-162 102)	43 489 (9194-169 142)	47 086 (9491-181 411)	43 167 (9055-171 676)	42 556 (9666-205 727)	47 260 (10 991-224 772)	12.8	<.001
**Specialists** ^c^										
Physicians as primary PI for NCEs receiving payments, No. (% of total)	14 877 (56.9)	14 648 (56.8)	14 424 (56.1)	14 258 (56.2)	13 320 (55.1)	12 677 (54.8)	12 273 (53.9)	11 439 (53.2)	−23.1	<.001
Payment value, million US $ (% of total)	2849.3 (63.1)	2829.9 (61.4)	2950.5 (61.8)	3021.8 (61.8)	2761.8 (61.0)	2741.2 (59.1)	2936.3 (53.0)	2794.5 (50.3)	−1.9	.61
Payment value per PI, median (IQR), US $	32 447 (7244-130 477)	32 794 (7542-135 333)	32 766 (7221-137 084)	34 268 (7654-145 676)	34 552 (7704-150 583)	34 802 (7779-145 156)	33 858 (7775-140 190)	35 372 (8234-140 602)	9.0	.88
**Surgeons** ^c^										
Physicians as primary PI for NCEs receiving payments, No. (% of total)	4882 (18.7)	4659 (18.1)	4556 (17.7)	4449 (17.5)	4489 (18.6)	4331 (18.7)	4316 (19.0)	4130 (19.2)	−15.4	<.001
Payment value, million US $ (% of total)	359.4 (8.0)	426.5 (9.3)	500.8 (10.5)	593.0 (12.1)	531.5 (11.7)	566.6 (12.2)	634.9 (11.5)	647.0 (11.6)	80.0	.001
Payment value per PI (IQR), US $	12 128 (2301-48 650)	19 095 (4136-72 198)	20 156 (4779-80 937)	20 952 (5282-73 052)	19 699 (4916-76 413)	19 577 (4722-67 404)	21 624 (5402-76 773)	20 847 (5677-77 272)	71.9	.001
**Interventionalists** ^c^										
Physicians as primary PI for NCEs receiving payments, No. (% of total)	1496 (5.7)	1543 (6.0)	1581 (6.2)	1606 (6.3)	1617 (6.7)	1479 (6.4)	1412 (6.2)	1289 (6.0)	−13.8	.09
Payment value, million US $ (% of total)	173.0 (3.8)	177.9 (3.9)	186.6 (3.9)	214.6 (4.4)	189.2 (4.2)	166.2 (3.6)	170.5 (3.1)	172.3 (3.1)	−0.4	.57
Payment value per PI (IQR), US $	24 230 (5974-87 828)	27 015 (6788-89 676)	27 464 (6565-86 008)	29 902 (8197-99 685)	27 183 (6879-92 925)	22 958 (6466-87 633)	20 813 (5859-81 056)	24 772 (6664-85 544)	2.2	.23

Abbreviations: NCE, noncovered entity; PI, principal investigator.

^a^ All payment values were inflation-adjusted to 2022 US $ values using the Consumer Price Index for All Urban Consumers: Medical Care in US City Average.

^b^ *P* values for yearly trends in physicians as the primary PI for NCEs receiving industry-sponsored research payments and payment values were obtained via linear regression. *P* values for yearly trends in payment values per physician PI were obtained via linear regression with a generalized estimating equation framework accounting for clustered physician effects and a γ link to account for positively skewed payment data.

^c^ See the eTable in Supplement 1 for definitions used for physician specialty groups.

## Discussion

This cohort study found that between 2015 and 2022, ISRPs increased by 20.0% to reach $7.58 billion by 2022. In comparison, National Institutes of Health research grants increased by 24% over this period, to $31.3 billion by 2022.^5^ Most ISRPs were directed to NCEs rather than teaching hospitals or physicians. While direct ISRPs to physicians steadily declined over time, ISRPs to NCEs with physician PIs increased to $5.55 billion by 2022, accounting for almost half of all Open Payments. General internists had the highest ISRPs per PI, potentially reflecting their access to patients with chronic diseases of interest for industry-sponsored pharmaceutical research.

Reporting rules in the PPSA specify that only the research entity, affiliated physician PIs, and total amount of the research payment are mandated for disclosure.^6^ Consequently, the industry is not obligated to divulge specific research payment amounts allocated to individual physicians within NCEs vs payments for direct research expenses. It becomes imperative to disclose ISRPs to individual physicians in NCEs to promote transparency and accountability within the health care system, as intended by the PPSA.

Our study has several limitations, including potential data reporting errors to the OPP, attribution of an ISRP exclusively to the primary PI, and unmeasured confounding. Despite these limitations, our study reveals a substantial increase in ISRPs to NCEs, comprising nearly half of overall Open Payments. Further research is needed to investigate factors associated with these payments to NCEs and their association with regulatory oversight and financial conflicts of interest.

## Notes

Supplement 1. **eMethods.** Recipient Definitions **eTable.** Taxonomic Codes for Physician Specialties and Definitions Used to Group Specialties

Supplement 2.
Data Sharing Statement
