# Supplementary material for: Trends in Industry-Sponsored Research Payments to Physician Principal Investigators
Source: JAMA Netw Open. 2024 May 16;7(5):e2412432. doi: 10.1001/jamanetworkopen.2024.12432 (PMC11099677; doi:10.1001/jamanetworkopen.2024.12432)
Supplement: Supplement 1. — eMethods. Recipient Definitions eTable. Taxonomic Codes for Physician Specialties and Definitions Used to Group Specialties [file jamanetwopen-e2412432-s001.pdf]

Supplemental Online Content

Su ZT, Hammadeh Z, Cheaib JG, Jing Y, Trock BJ, Han M. Trends in industry-sponsored research payments to physician principal investigators. *JAMA Netw Open*. 2024;7(5):e2412432. doi:10.1001/jamanetworkopen.2024.12432

eMethods. Recipient Definitions

eTable. Taxonomic Codes for Physician Specialties and Definitions Used to Group Specialties

This supplemental material has been provided by the authors to give readers additional information about their work.

© 2024 Su ZT et al. *JAMA Network Open.*

eMethods. Recipient Definitions

Definitions of recipients according to the Centers for Medicare and Medicaid Services (CMS) Open Payments program (https://openpaymentsdata.cms.gov/about/glossary).

Covered recipient: Any physician, physician assistant, nurse practitioner, clinical nurse specialist, certified registered nurse anesthetist, or certified nurse-midwife who is not a bona fide employee of the applicable manufacturer that is reporting the payment; or a teaching hospital, which is any institution that received a payment under 1886(d)(5)(B), 1886(h), or 1886(s) of the Act during the last calendar year for which such information is available.

Teaching hospital: For the purposes of Open Payments, “teaching hospitals” are hospitals that received payment for Medicare direct graduate medical education (GME), inpatient prospective payment system (IPPS) indirect medical education (IME), or psychiatric hospital IME programs during the last calendar year for which such information is available.

© 2024 Su ZT et al. *JAMA Network Open.*

Supplemental Table. Taxonomic Codes for Physician Specialties from the Centers for Medicare and Medicaid Services (CMS) Open Payments Covered Recipient Taxonomy List and Definitions Used to Group Specialties

Provider taxonomy code	Classification	Specialization	Specialty group definition
207KA0200X	Allergy & Immunology	Allergy	Specialists
207KI0005X	Allergy & Immunology	Clinical & Laboratory Immunology	Specialists
207K00000X	Allergy & Immunology		Specialists
207LA0401X	Anesthesiology	Addiction Medicine	Interventionalists
207LC0200X	Anesthesiology	Critical Care Medicine	Interventionalists
207LH0002X	Anesthesiology	Hospice and Palliative Medicine	Interventionalists
207LP2900X	Anesthesiology	Pain Medicine	Interventionalists
207LP3000X	Anesthesiology	Pediatric Anesthesiology	Interventionalists
207L00000X	Anesthesiology		Interventionalists
208U00000X	Clinical Pharmacology		Specialists
208C00000X	Colon & Rectal Surgery		Surgeons
207NI0002X	Dermatology	Clinical & Laboratory Dermatological Immunology	Specialists
207ND0900X	Dermatology	Dermatopathology	Specialists
207ND0101X	Dermatology	MOHS-Micrographic Surgery	Specialists
207NP0225X	Dermatology	Pediatric Dermatology	Specialists
207NS0135X	Dermatology	Procedural Dermatology	Specialists
207N00000X	Dermatology		Specialists
204R00000X	Electrodiagnostic Medicine		Specialists
207PE0004X	Emergency Medicine	Emergency Medical Services	Specialists
207PH0002X	Emergency Medicine	Hospice and Palliative Medicine	Specialists
207PT0002X	Emergency Medicine	Medical Toxicology	Specialists
207PP0204X	Emergency Medicine	Pediatric Emergency Medicine	Specialists
207PS0010X	Emergency Medicine	Sports Medicine	Specialists
207PE0005X	Emergency Medicine	Undersea and Hyperbaric Medicine	Specialists
207P00000X	Emergency Medicine		Specialists
207QA0401X	Family Medicine	Addiction Medicine	Specialists
207QA0000X	Family Medicine	Adolescent Medicine	General internists
207QA0505X	Family Medicine	Adult Medicine	General internists
207QG0300X	Family Medicine	Geriatric Medicine	General internists
207QH0002X	Family Medicine	Hospice and Palliative Medicine	Specialists
207QB0002X	Family Medicine	Obesity Medicine	Specialists
207QS1201X	Family Medicine	Sleep Medicine	Specialists
207QS0010X	Family Medicine	Sports Medicine	Specialists
207Q00000X	Family Medicine		General internists
208D00000X	General Practice		General internists

© 2024 Su ZT et al. *JAMA Network Open.*

Provider taxonomy code	Classification	Specialization	Specialty group definition
208M00000X	Hospitalist		General internists
202C00000X	Independent Medical Examiner		Specialists
202D00000X	Integrative Medicine		Specialists
207RA0401X	Internal Medicine	Addiction Medicine	Specialists
207RA0000X	Internal Medicine	Adolescent Medicine	General internists
207RA0002X	Internal Medicine	Adult Congenital Heart Disease	Specialists
207RA0001X	Internal Medicine	Advanced Heart Failure and Transplant Cardiology	Specialists
207RA0201X	Internal Medicine	Allergy & Immunology	Specialists
207RC0000X	Internal Medicine	Cardiovascular Disease	Specialists
207RI0001X	Internal Medicine	Clinical & Laboratory Immunology	Specialists
207RC0001X	Internal Medicine	Clinical Cardiac Electrophysiology	Specialists
207RC0200X	Internal Medicine	Critical Care Medicine	Specialists
207RE0101X	Internal Medicine	Endocrinology, Diabetes & Metabolism	Specialists
207RG0100X	Internal Medicine	Gastroenterology	Interventionalists
207RG0300X	Internal Medicine	Geriatric Medicine	Specialists
207RH0000X	Internal Medicine	Hematology	Specialists
207RH0003X	Internal Medicine	Hematology & Oncology	Specialists
207RI0008X	Internal Medicine	Hepatology	Specialists
207RH0002X	Internal Medicine	Hospice and Palliative Medicine	Specialists
207RH0005X	Internal Medicine	Hypertension Specialist	Specialists
207RI0200X	Internal Medicine	Infectious Disease	Specialists
207RI0011X	Internal Medicine	Interventional Cardiology	Interventionalists
207RM1200X	Internal Medicine	Magnetic Resonance Imaging (MRI)	Specialists
207RX0202X	Internal Medicine	Medical Oncology	Specialists
207RN0300X	Internal Medicine	Nephrology	Specialists
207RB0002X	Internal Medicine	Obesity Medicine	Specialists
207RP1001X	Internal Medicine	Pulmonary Disease	Specialists
207RR0500X	Internal Medicine	Rheumatology	Specialists
207RS0012X	Internal Medicine	Sleep Medicine	Specialists
207RS0010X	Internal Medicine	Sports Medicine	Specialists
207RT0003X	Internal Medicine	Transplant Hepatology	Specialists
207R00000X	Internal Medicine		General internists
209800000X	Legal Medicine		Specialists
207SG0202X	Medical Genetics	Clinical Biochemical Genetics	Specialists
207SC0300X	Medical Genetics	Clinical Cytogenetic	Specialists
207SG0201X	Medical Genetics	Clinical Genetics (M.D.)	Specialists
207SG0203X	Medical Genetics	Clinical Molecular Genetics	Specialists
207SM0001X	Medical Genetics	Molecular Genetic Pathology	Specialists
207SG0205X	Medical Genetics	Ph.D. Medical Genetics	Specialists
207T00000X	Neurological Surgery		Surgeons

© 2024 Su ZT et al. *JAMA Network Open.*

Provider taxonomy code	Classification	Specialization	Specialty group definition
204D00000X	Neuromusculoskeletal Medicine & OMM		Specialists
204C00000X	Neuromusculoskeletal Medicine, Sports Medicine		Specialists
207UN0903X	Nuclear Medicine	In Vivo & In Vitro Nuclear Medicine	Specialists
207UN0901X	Nuclear Medicine	Nuclear Cardiology	Specialists
207UN0902X	Nuclear Medicine	Nuclear Imaging & Therapy	Specialists
207U00000X	Nuclear Medicine		Specialists
207VC0300X	Obstetrics & Gynecology	Complex Family Planning	Surgeons
207VC0200X	Obstetrics & Gynecology	Critical Care Medicine	Surgeons
207VF0040X	Obstetrics & Gynecology	Female Pelvic Medicine and Reconstructive Surgery	Surgeons
207VX0201X	Obstetrics & Gynecology	Gynecologic Oncology	Surgeons
207VG0400X	Obstetrics & Gynecology	Gynecology	Surgeons
207VH0002X	Obstetrics & Gynecology	Hospice and Palliative Medicine	Surgeons
207VM0101X	Obstetrics & Gynecology	Maternal & Fetal Medicine	Surgeons
207VB0002X	Obstetrics & Gynecology	Obesity Medicine	Surgeons
207VX0000X	Obstetrics & Gynecology	Obstetrics	Surgeons
207VE0102X	Obstetrics & Gynecology	Reproductive Endocrinology	Surgeons
207V00000X	Obstetrics & Gynecology		Surgeons
207WX0120X	Ophthalmology	Cornea and External Diseases Specialist	Surgeons
207WX0009X	Ophthalmology	Glaucoma Specialist	Surgeons
207WX0109X	Ophthalmology	Neuro-ophthalmology	Surgeons
207WX0200X	Ophthalmology	Ophthalmic Plastic and Reconstructive Surgery	Surgeons
207WX0110X	Ophthalmology	Pediatric Ophthalmology and Strabismus Specialist	Surgeons
207WX0107X	Ophthalmology	Retina Specialist	Surgeons
207WX0108X	Ophthalmology	Uveitis and Ocular Inflammatory Disease	Surgeons
207W00000X	Ophthalmology		Surgeons
204E00000X	Oral & Maxillofacial Surgery		Surgeons
207XS0114X	Orthopaedic Surgery	Adult Reconstructive Orthopaedic Surgery	Surgeons
207XX0004X	Orthopaedic Surgery	Foot and Ankle Surgery	Surgeons
207XS0106X	Orthopaedic Surgery	Hand Surgery	Surgeons
207XS0117X	Orthopaedic Surgery	Orthopaedic Surgery of the Spine	Surgeons
207XX0801X	Orthopaedic Surgery	Orthopaedic Trauma	Surgeons
207XP3100X	Orthopaedic Surgery	Pediatric Orthopaedic Surgery	Surgeons
207XX0005X	Orthopaedic Surgery	Sports Medicine	Surgeons
207X00000X	Orthopaedic Surgery		Surgeons
207YS0123X	Otolaryngology	Facial Plastic Surgery	Surgeons
207YX0602X	Otolaryngology	Otolaryngic Allergy	Surgeons
207YX0905X	Otolaryngology	Otolaryngology/Facial Plastic Surgery	Surgeons
207YX0901X	Otolaryngology	Otology & Neurotology	Surgeons

© 2024 Su ZT et al. *JAMA Network Open.*

Provider taxonomy code	Classification	Specialization	Specialty group definition
207YP0228X	Otolaryngology	Pediatric Otolaryngology	Surgeons
207YX0007X	Otolaryngology	Plastic Surgery within the Head & Neck	Surgeons
207YS0012X	Otolaryngology	Sleep Medicine	Surgeons
207Y00000X	Otolaryngology		Surgeons
208VP0014X	Pain Medicine	Interventional Pain Medicine	Specialists
208VP0000X	Pain Medicine	Pain Medicine	Specialists
207ZP0101X	Pathology	Anatomic Pathology	Specialists
207ZP0102X	Pathology	Anatomic Pathology & Clinical Pathology	Specialists
207ZB0001X	Pathology	Blood Banking & Transfusion Medicine	Specialists
207ZP0104X	Pathology	Chemical Pathology	Specialists
207ZC0008X	Pathology	Clinical Informatics	Specialists
207ZC0006X	Pathology	Clinical Pathology	Specialists
207ZP0105X	Pathology	Clinical Pathology/Laboratory Medicine	Specialists
207ZC0500X	Pathology	Cytopathology	Specialists
207ZD0900X	Pathology	Dermatopathology	Specialists
207ZF0201X	Pathology	Forensic Pathology	Specialists
207ZH0000X	Pathology	Hematology	Specialists
207ZI0100X	Pathology	Immunopathology	Specialists
207ZM0300X	Pathology	Medical Microbiology	Specialists
207ZP0007X	Pathology	Molecular Genetic Pathology	Specialists
207ZN0500X	Pathology	Neuropathology	Specialists
207ZP0213X	Pathology	Pediatric Pathology	Specialists
2080A0000X	Pediatrics	Adolescent Medicine	General internists
2080C0008X	Pediatrics	Child Abuse Pediatrics	Specialists
2080I0007X	Pediatrics	Clinical & Laboratory Immunology	Specialists
2080P0006X	Pediatrics	Developmental – Behavioral Pediatrics	Specialists
2080H0002X	Pediatrics	Hospice and Palliative Medicine	Specialists
2080T0002X	Pediatrics	Medical Toxicology	Specialists
2080N0001X	Pediatrics	Neonatal-Perinatal Medicine	Specialists
2080P0008X	Pediatrics	Neurodevelopmental Disabilities	Specialists
2080B0002X	Pediatrics	Obesity Medicine	Specialists
2080P0201X	Pediatrics	Pediatric Allergy/Immunology	Specialists
2080P0202X	Pediatrics	Pediatric Cardiology	Specialists
2080P0203X	Pediatrics	Pediatric Critical Care Medicine	Specialists
2080P0204X	Pediatrics	Pediatric Emergency Medicine	Specialists
2080P0205X	Pediatrics	Pediatric Endocrinology	Specialists
2080P0206X	Pediatrics	Pediatric Gastroenterology	Interventionalists
2080P0207X	Pediatrics	Pediatric Hematology-Oncology	Specialists
2080P0208X	Pediatrics	Pediatric Infectious Diseases	Specialists
2080P0210X	Pediatrics	Pediatric Nephrology	Specialists

© 2024 Su ZT et al. *JAMA Network Open.*

Provider taxonomy code	Classification	Specialization	Specialty group definition
2080P0214X	Pediatrics	Pediatric Pulmonology	Specialists
2080P0216X	Pediatrics	Pediatric Rheumatology	Specialists
2080T0004X	Pediatrics	Pediatric Transplant Hepatology	Specialists
2080S0012X	Pediatrics	Sleep Medicine	Specialists
2080S0010X	Pediatrics	Sports Medicine	Specialists
208000000X	Pediatrics		General internists
202K00000X	Phlebology		Specialists
2081P0301X	Physical Medicine & Rehabilitation	Brain Injury Medicine	Specialists
2081H0002X	Physical Medicine & Rehabilitation	Hospice and Palliative Medicine	Specialists
2081N0008X	Physical Medicine & Rehabilitation	Neuromuscular Medicine	Specialists
2081P2900X	Physical Medicine & Rehabilitation	Pain Medicine	Specialists
2081P0010X	Physical Medicine & Rehabilitation	Pediatric Rehabilitation Medicine	Specialists
2081P0004X	Physical Medicine & Rehabilitation	Spinal Cord Injury Medicine	Specialists
2081S0010X	Physical Medicine & Rehabilitation	Sports Medicine	Specialists
208100000X	Physical Medicine & Rehabilitation		Specialists
2082S0099X	Plastic Surgery	Plastic Surgery Within the Head and Neck	Surgeons
2082S0105X	Plastic Surgery	Surgery of the Hand	Surgeons
208200000X	Plastic Surgery		Surgeons
2083A0300X	Preventive Medicine	Addiction Medicine	Specialists
2083A0100X	Preventive Medicine	Aerospace Medicine	Specialists
2083C0008X	Preventive Medicine	Clinical Informatics	Specialists
2083T0002X	Preventive Medicine	Medical Toxicology	Specialists
2083B0002X	Preventive Medicine	Obesity Medicine	Specialists
2083X0100X	Preventive Medicine	Occupational Medicine	Specialists
2083P0500X	Preventive Medicine	Preventive Medicine/Occupational Environmental Medicine	Specialists
2083P0901X	Preventive Medicine	Public Health & General Preventive Medicine	Specialists
2083S0010X	Preventive Medicine	Sports Medicine	Specialists
2083P0011X	Preventive Medicine	Undersea and Hyperbaric Medicine	Specialists
2084A0401X	Psychiatry & Neurology	Addiction Medicine	Specialists
2084P0802X	Psychiatry & Neurology	Addiction Psychiatry	Specialists
2084B0040X	Psychiatry & Neurology	Behavioral Neurology & Neuropsychiatry	Specialists
2084P0301X	Psychiatry & Neurology	Brain Injury Medicine	Specialists
2084P0804X	Psychiatry & Neurology	Child & Adolescent Psychiatry	Specialists
2084N0600X	Psychiatry & Neurology	Clinical Neurophysiology	Specialists
2084D0003X	Psychiatry & Neurology	Diagnostic Neuroimaging	Specialists
2084E0001X	Psychiatry & Neurology	Epilepsy	Specialists
2084F0202X	Psychiatry & Neurology	Forensic Psychiatry	Specialists
2084P0805X	Psychiatry & Neurology	Geriatric Psychiatry	Specialists
2084H0002X	Psychiatry & Neurology	Hospice and Palliative Medicine	Specialists

© 2024 Su ZT et al. *JAMA Network Open.*

Provider taxonomy code	Classification	Specialization	Specialty group definition
2084A2900X	Psychiatry & Neurology	Neurocritical Care	Specialists
2084P0005X	Psychiatry & Neurology	Neurodevelopmental Disabilities	Specialists
2084N0400X	Psychiatry & Neurology	Neurology	Specialists
2084N0402X	Psychiatry & Neurology	Neurology with Special Qualifications in Child Neurology	Specialists
2084N0008X	Psychiatry & Neurology	Neuromuscular Medicine	Specialists
2084B0002X	Psychiatry & Neurology	Obesity Medicine	Specialists
2084P2900X	Psychiatry & Neurology	Pain Medicine	Specialists
2084P0800X	Psychiatry & Neurology	Psychiatry	Specialists
2084P0015X	Psychiatry & Neurology	Psychosomatic Medicine	Specialists
2084S0012X	Psychiatry & Neurology	Sleep Medicine	Specialists
2084S0010X	Psychiatry & Neurology	Sports Medicine	Specialists
2084V0102X	Psychiatry & Neurology	Vascular Neurology	Specialists
2085B0100X	Radiology	Body Imaging	Specialists
2085D0003X	Radiology	Diagnostic Neuroimaging	Specialists
2085R0202X	Radiology	Diagnostic Radiology	Specialists
2085U0001X	Radiology	Diagnostic Ultrasound	Specialists
2085H0002X	Radiology	Hospice and Palliative Medicine	Specialists
2085N0700X	Radiology	Neuroradiology	Specialists
2085N0904X	Radiology	Nuclear Radiology	Specialists
2085P0229X	Radiology	Pediatric Radiology	Specialists
2085R0001X	Radiology	Radiation Oncology	Specialists
2085R0205X	Radiology	Radiological Physics	Specialists
2085R0203X	Radiology	Therapeutic Radiology	Specialists
2085R0204X	Radiology	Vascular & Interventional Radiology	Specialists
2086H0002X	Surgery	Hospice and Palliative Medicine	Surgeons
2086S0120X	Surgery	Pediatric Surgery	Surgeons
2086S0122X	Surgery	Plastic and Reconstructive Surgery	Surgeons
2086S0105X	Surgery	Surgery of the Hand	Surgeons
2086S0102X	Surgery	Surgical Critical Care	Surgeons
2086X0206X	Surgery	Surgical Oncology	Surgeons
2086S0127X	Surgery	Trauma Surgery	Surgeons
2086S0129X	Surgery	Vascular Surgery	Surgeons
208600000X	Surgery		Surgeons
208G00000X	Thoracic Surgery (Cardiothoracic Vascular Surgery)		Surgeons
204F00000X	Transplant Surgery		Surgeons
2088F0040X	Urology	Female Pelvic Medicine and Reconstructive Surgery	Surgeons
2088P0231X	Urology	Pediatric Urology	Surgeons
208800000X	Urology		Surgeons

© 2024 Su ZT et al. *JAMA Network Open.*
