# Supplementary material for: Trends in Industry-Sponsored Research Payments to Physician Principal Investigators
Source: JAMA Netw Open. 2024 May 16;7(5):e2412432. doi: 10.1001/jamanetworkopen.2024.12432 (PMC11099677; doi:10.1001/jamanetworkopen.2024.12432)
Supplement: Supplement 2. — Data Sharing Statement [file jamanetwopen-e2412432-s002.pdf]

Data Sharing Statement

Su. Trends in Industry-Sponsored Research Payments to Physician Principal Investigators. *JAMA Netw Open*. Published online May 16, 2024. doi:10.1001/jamanetworkopen.2024.12432

Data
Data available: Yes
Data types: Data (not involving human participants)
How to access data: Our study used only publicly available data from the Centers for Medicare and Medicaid Services (CMS) Open Payments program, with all data freely accessible from: https://openpaymentsdata.cms.gov/.
When available: With publication

Supporting Documents
Document types: Other (please specify)
Additional Information: Supplemental Table included with the manuscript submission, to include definitions used to group physician specialties into 4 categories in our data analyses
How to access documents: Supplemental Table as part of the manuscript submission
When available: With publication

Additional Information
Who can access the data: https://openpaymentsdata.cms.gov/
Types of analyses: For any purpose
Mechanisms of data availability: Without investigator support
Any additional restrictions: N/A
